# Food literacy competencies in youth – a mini-review

**DOI:** 10.3389/fpubh.2023.1185410

**Published:** 2023-07-20

**Authors:** Synne Groufh-Jacobsen, Anine Christine Medin

**Affiliations:** Department of Nutrition and Public Health, Faculty of Health and Sports Sciences, University of Agder, Kristiansand, Norway

**Keywords:** food literacy, nutrition literacy, critical nutrition literacy, nutrition knowledge, food skills, youth, public health

## Abstract

**Introduction:**

Young people’s transition into adulthood is an opportunity in the life course to establish adequate eating behaviors, hence exploring food literacy competencies in this period of life is especially important. Food literacy has recently gained increased attention in adults, adolescents, and younger children, but less is published about youth. This paper aims to summarize what tools have been used to measure food literacy and the sub-competence nutrition literacy in youth aged 16–24 years in the previous 5 years.

**Methods:**

A mini-literature review was conducted in MEDLINE and EMBASE via Ovid, in September 2022. Study eligible criteria; had to be an original article, using a tool to quantitatively assess food literacy and/or nutrition literacy, including participants between 16–24 years, full text available in English, published between 2017–2022.

**Results:**

A total of 958 articles were identified, of which 385 duplicates were removed. Thus, 573 articles were screened by title/abstract. Finally, nine articles were eligible for data extraction of which four proposed a tool to measure food literacy and five proposed a tool to measure nutrition literacy.

**Discussion and conclusion:**

Although four studies claimed to measure food literacy, none of these used tools comprehensive enough to measure all aspects of food literacy, and only one was validated in young people. This study shows that only few tools exist for the measurement of food literacy in youth, and those available are scant. Further work is needed to develop a food literacy tool for youth.

## Highlights

- This mini-review aims to summarize what tools have been used to measure food literacy and the sub-competence nutrition literacy in youth aged 16–24 years in the previous five years.
- A mini-literature review was conducted in MEDLINE and EMBASE via Ovid, in September 2022 and the literature search revealed that comprehensive tools measuring food literacy in youth are lacking.
- Continued effort to achieve consensus on how to measure food literacy in youth is needed.

## 1. Introduction

Youth is a period in life where many become more responsible for what to eat, when to eat, and how to eat (1). At the same time, the food landscape is complex and rapidly evolving, which makes it a challenge for the individual consumer to make food choices that ensure a healthy diet. Unhealthy eating habits and poor diet quality over time can increase the risk of malnutrition and later in life increase the risk of non-communicable diseases. Hence it is crucial to establish healthy eating habits early in life and during the life course, especially in the transition to adulthood (2). The concept of food literacy is increasingly applied in the academic literature, especially in adults (3–6), and several definitions of food literacy exist (3, 5–10).

The food literacy definition proposed by Vidgen & Gallegos is the most cited definition for measuring food literacy; it consists of 11 components within the competencies of planning and managing, selecting, preparing, and eating foods (10, 11). Vidgen & Gallegos define food literacy as “a collection of interrelated knowledge, skills and behaviors required to plan, manage, select, prepare and eat foods to meet needs and determine food intake” and as “the scaffolding that empowers individuals, households, communities or nations to protect diet quality” (5). Nutrition literacy is suggested as a sub-competence of food literacy (8). Nutrition literacy is a construct consisting of three sub-levels derived from the health literacy framework by Nutbeam (12). Nutrition literacy is based on the same three sub-levels as health literacy but is used in a nutritional context (8, 13). In a previous study, it was reported that experts in the field have agreed upon the definition of food literacy by Vidgen & Gallegos for measuring food literacy in adults (11). A new tool for measuring food literacy in adults has been proposed based on the definition by Vidgen & Gallegos, however, there is still disagreement on which items should be included to capture the food literacy competencies in an international context, e.g., cultural considerations (14). More work is also needed to establish a tool for measuring food literacy in youth. Slater and coworkers (1) have proposed an even broader food literacy framework for youths than the Vidgen & Gallegos definition, and suggested that youth require much more than basic nutrition knowledge and food skills to navigate the complex food environment. Currently, no tool exists based on the suggested food literacy framework by Slater et al. According to Slater et al., and Vettori, et al., food literacy involves not only individual abilities, but also social, environmental, political, cultural, and economic aspects of food behavior (1, 9).

Previous reviews have summed up which aspects of food literacy are measured in the literature and what tools are being used in children (2–12 years), adolescents (13–18 years) (4) and adults (3, 8, 15). However, no previous review has emphasized tools used to measure food literacy in youth (16), which is the period between adolescence and adulthood. Thus, an overview of what tools have been used to measure food literacy in youth is lacking. This mini-review responds to this and aims to summarize tools used to measure food literacy and nutrition literacy (sub-competence of food literacy) in youths 16–24 years in the previous five years.

## 2. Method

For this mini-review, the following eligibility criteria were used to evaluate articles for data extraction. To be included, the article had to be an original article, presenting separate data in the age group 16–24 years, using a tool to quantitatively assess food literacy and/or nutrition literacy (sub-competence of food literacy), available in full text in the English language, and published between 2017–2022. The previous five years were used as eligibility criteria to give an insight into the tools currently being used. Articles were excluded if being qualitative studies due to lack of a tool that quantitatively assesses food literacy and/or nutrition literacy. Further, studies were excluded if they lacked information concerning the tool/questionnaire used to measure food literacy or nutrition literacy. Additionally, articles being part of the school curriculum or measuring general nutrition knowledge/sports nutrition knowledge or measuring nutrition knowledge related to a specific food/or behavior were excluded.

### 2.1. Literature search and screening process

**Figure 1:**
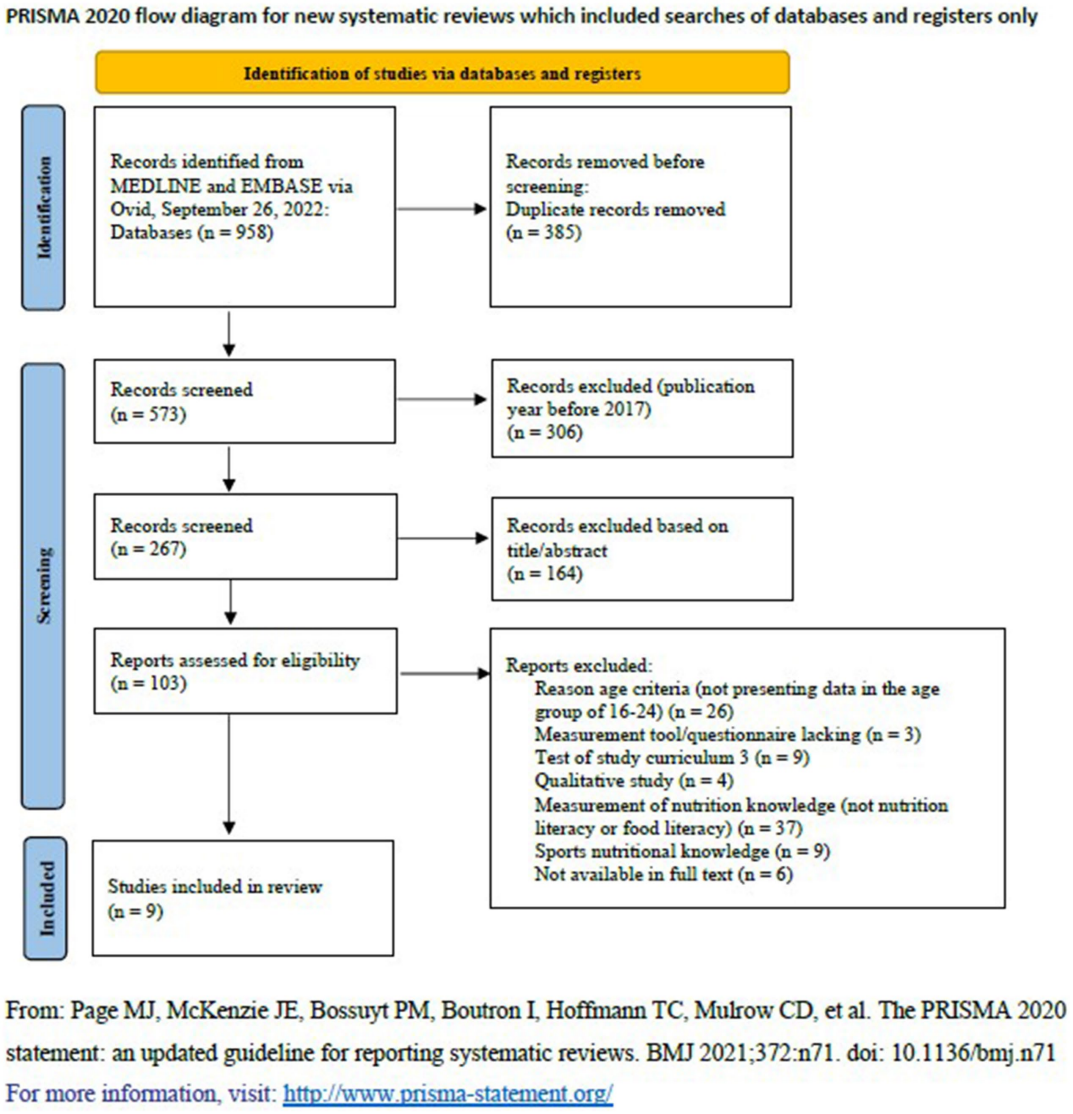
Literature search and screening process.

The literature search and screening process is presented in Figure 1. A systematic search and a comprehensive review of the literature were performed on September 26, 2022, in MEDLINE and EMBASE via Ovid. The following keywords were used: (1) “(youth OR juvenile OR adolescen* OR young* OR teenage* OR teens OR student*).ti. (2) ((nutrition* OR food* OR diet*) adj3 (knowledge* OR competence* OR literac*).ti,ab. (3) limit 6 to yr. = “2010-Current” (4) (nutrition* OR food* OR diet*) and knowledge* OR competence* OR literac*).ti. (5) NOT (review* OR meta-analys* OR systematic review.ti. Google Scholar was searched on September 26, 2022, using similar keywords to ensure that all published articles on the topic were screened.

The authors discussed and agreed on the eligibility criteria prior to the literature search, and the literature search was performed by the first author, with the assistance of a librarian. The first author performed all parts of the screening.

In the study by Liao et al. (17) mean age of the participants was not reported. However, the authors reported to include first, second, third- and fourth-year Taiwan university students. This study was included based on the reported age of Taiwan university students in another included study by Lai et al. (mean (SD) age of 20 ± 2) (18). Hence, we assume that the participants in the study by Liao et al., are in our target group (16–24 years).

## 3. Results

The literature search resulted in 958 articles, of which 385 were duplicates and removed. In total, 573 articles were screened by publication year, of which 306 articles were excluded due to being published before 2017. For the second screening, 267 articles were screened by title and abstract, of which 164 articles were excluded based on eligibility criteria. For full-text screening, 103 articles were screened and read to confirm study eligibility, out of which nine articles were eligible for data extraction in this mini-review (Figure 1).

The data extraction from the included literature in this mini-review is presented in Table 1. The studies included describes tools published between 2017–2022, in which four studies (three different tools) measured food literacy (19, 21, 23, 24) and five studies measured nutrition literacy or sub-dimensions of nutrition literacy (17, 18, 25, 26, 28). The mean age in the included studies ranged from 17–24 years, except for one previously mentioned study that did not report mean age (17). The purpose of most of the studies was to either assess the level of food literacy or nutrition literacy of young people with already existing tools, while four of the studies had the purpose to develop and test the validity and/or reliability of a newly developed tool (21, 23, 25, 28).

**Table 1:** Literature list used for data extraction.

Authors/years	Study population	Measurement tool	Concept to be measured
Ashoori et al. (19)^a^	Iranian senior high school students, mean ± SD age, 17.8 ± 0.4 (*n* = 621).	The food and Nutrition Literacy assessment tool (FNLAT) consists of 60 items (20) of which 30 items with dichotomous answering and 30 items with Likert-type statements. The FNLAT tool is divided into six dimensions: Food and nutrition knowledge (27 items), functional skills (11 items), interactive skills (7 items), advocacy (7 items), critical analysis of information (5 items), and food label reading skills (3 items). FNL score ranged from 0–100.	Food and nutrition literacy
Durmus et al. (21)^b^	Harran University School, mean ± SD age, 19.9 ± 2.4 (*n* = 308).	Short Food Literacy Questionnaire (SFLQ) developed by Krause et al. (22). The SFLQ tool consists of twelve items arranged on a four-or five-point Likert type scale. SFLQ score ranged from 7–52.	Food literacy
Na and Cho. (23)^b^	Young Korean people, mean ± SD age, 24.0 ± 2.6 (*n* = 435)	Food literacy tool developed for adult Korean people based on eleven food literacy. Components in a scoping review (6) (knowledge, food skills, food choice, self-efficacy, meal management, food safety, food security, food systems, food resource management, emotions, and sociocultural context). The final tool consisted of 50 items divided into two domains (food and nutrition knowledge and meal management) with 25 items assessing each domain.	Food literacy
Itzkovitz et al. (24)^a^	Canadian adults, people living with type 1 diabetes (*n* = 236) mean ± SD age 24.3 (3.3), and the control group 22.5 ± 3.4 (*n* = 191)	Short Food Literacy Questionnaire (SFLQ) developed by Krause et al., (22). The SFLQ tool was adapted to the 2007 Canadian Food guideline. The SFLQ tool consists of twelve items arranged on a four-or five-point Likert type scale. SFLQ score ranged from 7–52. Cooking skills were assessed by one item regarding the ability to cook with six answer options: - “I do not know where to start when cooking” - “I can do things such as boil an egg or cook a grilled cheese - “I can prepare simple meals but nothing too complicated. - “I can prepare most dishes” - “I can cook most dishes if I have a recipe to follow” - “I frequently prepare sophisticated dishes”	Food and nutrition literacy Cooking skills (not included in the SFLQ tool)
McNamara et al. (25)^b^	Students from the University of Maine and Rutgers. Between the age of 18–24 mean ± SD age of 19.9 ± 1.8 (*n* = 672).	Nutrition literacy tool consisting of 67 items divided into three subsections (functional, interactive, critical) based on emerging themes in focus groups and on a previously validated critical nutrition literacy tool (13). The items were arranged on a five-point Likert scale ranging from strongly disagree to strongly agree.	Nutrition literacy - Functional - Interactive - Critical skills
Lai et al. (18)^a^	University students in Taiwan, mean ± SD age at 20.12 ± 1.8 (*n* = 412).	Eighth-item nutrition literacy tool. Likert-type statements. Divided into five dimensions, obtaining nutritional information (2 items), understating basic nutrition information (2 items), analyzing different types of nutrition information (1 item), apprise and ability to judge and assess nutritional information (2 items) and the capacity to apply nutrition information (1 item).	Nutrition literacy - Functional - Interactive - Critical skills
Liao and Chang (17)^a^	University students in Taiwan (*n* = 119). Separate data was presented for first, second, third- and fourth-year university students.	Self-rated nutrition literacy tool. Divided into five domains of nutrition literacy: obtain nutrition information (2 items) understand nutrition information (2 items), analyze nutrition information (1 item), apprise nutrition information (1 item), and apply nutrition information (2 items). The response options were based on a four-point Likert-type scale.	Nutrition literacy - Functional - Interactive - Critical skills
Yilmazel and Bozdogan (26)^a^	Turkish adolescents. The total study sample consisted of 307 participants in the age range of 14–19. Separate results are presented for the age group 17–19 (*n* = 173).	The nutrition literacy scale was based on a previously developed tool (27), consisting of 22 items. Divided into three sub-dimensions (functional 7 items, interactive 6 items, and critical literacy 9 items). Items were arranged on a five-point Likert-type scale. The score ranged from 22–110 as the maximum.	Nutrition literacy - Functional - Interactive - Critical skills
Bedoyan et al. (28)^b^	US college students between 18–24, mean ± SD age at 18.4 ± 1.0 (*n* = 50).	Critical Nutrition Literacy Tool (CNLT-R) based on a previously validated tool by Guttersrud et al., (13). The CNLT-R tool consisted of seven items. Arranged on a five-point Likert scale ranging from strongly disagree to strongly agree.	Nutrition literacy - Critical skills

a Study design: cross-sectional.

b Study design: validation study.

### 3.1. Tools used to measure food literacy

In the study by Ashoori, M. et al., food literacy was measured using a previously validated tool for youths consisting of 60 items, the Food and Nutrition Literacy Assessment Tool (FNLAT) (19, 20). The FNLAT tool used by Ashoori, M, and coworkers is based on the health literacy definition by Nutbeam (12, 20). The FNLAT tool is divided into two domains, knowledge, and skills. The FNLAT tool also assesses sub-competencies within these two domains (food and nutrition knowledge, functional skills, interactive skills, advocacy, critical analysis of information, and food label reading skills) (19).

Two of the included studies that intended to measure food literacy aimed to test the reliability and validity of a food literacy tool in the target population 16–24 years (21, 23). The study by Durmus et al. (21) intended to adapt and validate the short food literacy questionnaire (SFLQ) tool developed by Krause et al. (22). The SFLQ tool focuses on individual skills and abilities needed for making healthy food choices and the tool is based on the definition of health literacy by Nutbeam (12). The authors that developed the SFLQ tool underline that the tool does not capture all aspects of food literacy (22) and refer to the definition by Vidgen et al. (5), and emphasize that the SFLQ tool is intended to be a rapid and practical tool for measuring food literacy in adults. The SFLQ tool consists of 12 items divided into two domains (food and nutrition knowledge and meal management). The SFLQ tool was used in two of the included studies for measurement of food literacy (21, 24). In the study by Itzkovitz et al., the SFLQ tool was used to measure the nutrition, health, and food literacy in adults living with type 1 diabetes compared to a healthy control group (24).

In the study by Na and Cho (23), a three-phase process was conducted to develop a tool for measuring food literacy in Koreans. The final tool consisted of 50 items divided into two domains, food, and nutrition knowledge (25 items) and meal management (25 items). The items were based on the 11 components of food literacy identified in a previous scoping review (6).

### 3.2. Tools used to measure nutrition literacy (sub-competence of food literacy)

Most of the studies referred to nutrition literacy as a construct with three sublevels (functional, interactive, and critical nutrition literacy) (18, 25, 26, 28) and considered to be a subset of health literacy (12). Four of the nine included studies in this mini-review used a tool that measured all three sublevels of nutrition literacy (17, 18, 25, 26), while one of the included studies measured only two subset of nutrition literacy (functional and critical nutrition literacy) (28).

Tools used to measure all three sublevels of nutrition literacy differed. One tool consisted of eight items divided into five domains (obtaining, understanding, analyzing, assessing, and applying nutrition information). The eight-item nutrition literacy tool was used in two of the included studies (17, 18). One of the other included studies that measured all three sublevels of nutrition literacy consists of 22 items divided into three subsections (functional, interactive, critical) (26) and referred to the definition by Guttersrud et al. (29) and Velardo (7). Both the eight-item tool and the 22-item tool referred to nutrition literacy as a subset of health literacy based on the definition by Nutbeam (12). In contrast, one of the other included studies that measured all three sublevels suggested using a more comprehensive tool consisting of 67 items divided into three sublevels (functional, interactive, critical) (25) and the study referred to the definition by Velardo (7).

The study by Bedoyan et al. (28) used a critical nutrition literacy tool by Guttersrud et al. (13) and aimed to establish the criterion validity of a revised version of the critical nutrition literacy tool in a US population. The study referred to both the definition by Velardo and Nutbeam (7, 12). The original tool consisted of two scales (engagement scale and claims scale). The revised critical nutrition literacy tool included the claims scale of which seven of the 11 items were included.

## 4. Discussion and conclusion

The understanding of the concept of food literacy is evolving (1, 5–7, 10, 11). Tools used to measure food literacy or sub-competencies of food literacy have previously been emphasized among children (3–6 years) (4), adolescents (9–18 years) (4) (10–19 years) (30) and adults (8, 14, 15, 31). To our knowledge, this mini-review is the first to summarize existing tools used to evaluate food literacy and nutrition literacy targeting youth (16–24 years). Out of the nine included studies in this mini-review, four tools measured food literacy, and five tools measured nutrition literacy or sub-dimensions of nutrition literacy.

Measurement of food literacy was assessed in four of the included studies (19, 21, 23, 24), in which two of the studies used the SFLQ tool based on the health literacy definition by Nutbeam (21, 24). The SFLQ tool was originally developed by Krause (22) as a feasible and reliable tool for the assessment of food literacy in adults, and the authors emphasize that the tool did not intend to measure all suggested aspects of food literacy by Vidgen and Gallegos. The SFLQ tool is only able to indicate key elements of food literacy and may be used in public health surveys wanting to increase the food literacy focus, however, this tool cannot be used to measure the whole concepts of food literacy as described by Vidgen and Gallegos (5). Thus, several of the competencies within the areas ‘functional competencies’, ‘relational competencies’ and ‘system competencies’ in the food literacy framework suggested for youth (1) is underrepresented in the SFLQ tool.

More comprehensive food literacy tools were also identified, the FNLAT tool consisting of 60 items (19), and a food literacy tool developed for Korean adults, consisting of 50 items (23). The FNLAT tool is based on the Nutbeam framework for health literacy (functional, interactive, and critical skills). Food and nutrition knowledge was included in the FNLAT tool in addition to the skills section, as having basic nutrition knowledge was reported to be an important component of food and nutrition literacy in the literature (8). The original authors who developed the FNLAT tool underline that the FNLAT tool is not designed to measure all aspects of food literacy, especially not food skills (20). Thus, the FNLAT tool might not sufficiently cover aspects of the ‘functional competencies’ as ‘food preparation skills’, ‘food budgeting skills’, ‘food & hygiene knowledge’ and ‘be able to think critically about and act on food and nutrition issues’, and the areas ‘relational competencies’ and ‘system competencies is lacking’, which are areas in the suggested food literacy framework for youth by Slater et al. (1).

The 50-item food literacy tool developed for Korean adults was the only tool based on food literacy aspects identified in a scoping review and not based on the health literacy definition by Nutbeam. The 50-item tool includes the food literacy aspects food knowledge, food safety, food systems, sociocultural context, food skills, food choices, food resource management, and self-efficacy. However, several of the items are related to Korean food practices, which may not apply to other countries with other food practices, and it is developed for assessing food literacy in adults. Therefore, it is possible that this tool may not adequately encompass critical aspects of the ‘functional competencies’ suggested for youth by Slater, including ‘have a healthy food relationship’ and ‘be able to think critically about and act on food and nutrition issues’, ‘food skills’ and ‘basic nutritional knowledge’.‘Relational competencies’ is also lacking in the tool (1).

To adapt a food literacy tool for a new population, it is crucial to consider cultural sensitivity and to conduct pilot testing and validation for accuracy and suitability for the target population. Moreover, researchers who develop a new tool should provide information on how others can adapt the tool to different cultures, e.g., using national dietary guidelines to assess general nutritional knowledge. Additionally, future research should prioritize making tools available in both the original language and English to enhance transparency and accessibility for other researchers.

None of the identified tools can be used in an international context nor are they comprehensive enough to capture and measure the whole concept of food literacy in youth. It has previously been emphasized that youth may require more basic nutrition knowledge and food skills compared to adults (1), as they are in a transition phase to adulthood. The transition phase into adulthood requires more responsibilities in regard to education, family, employment, living agreement and securing a healthy diet. Having sufficient food literacy competencies is important for youth to be able to secure a diet in alignment with the dietary guidelines, and to avoid a diet that will inflict implications over the life course (1). Adapting existing food literacy tools validated in an adult population to a youth population may pose potential challenges. When adapting a tool to a new target population, several aspects must be considered, such as age-specificity, language-appropriateness, and cultural sensitivity, to ensure its suitability. It is also crucial to pilot test and assess the validity of the adapted tool to ensure its accuracy for the new target population. The SFLQ tool and the 50-item food literacy tool for Koreans were developed for adults, only the FNLAT tool was developed for younger people. Most tools measuring nutrition literacy included in this mini-review were originally developed for adults, despite being used in younger age groups.

A consensus on how to define and measure the concept of food literacy in youths is needed, including the sub-competencies described in the comprehensive food literacy framework by Slater (1). However, given that the framework comprises 16 broad competencies areas and 59 specific competencies, it may be challenging to develop a single tool that covers all aspects of food literacy as defined by the framework. This is evident by the fact that none of the identified studies in this mini– review used a tool covering the framework. Continued work and effort are therefore needed to develop a tool that assesses food literacy in youth, as there is still a substantial gap between how food literacy currently is being assessed in youth today and the competencies covered by the broader framework suggested for this age group (1).

Findings from this mini-review are limited to food literacy tools published after 2017 to give a brief up-to-date on tools currently being used. We may therefore not have identified all available tools measuring food literacy in the target population. Another limitation is that only articles available in the English language were included, which may have resulted in excluding relevant tools available in other languages. Another limitation was that the screening process was not blinded and performed by one person, however the eligibility criteria were discussed by the authors prior to the screening process. Overall, these limitations need to be considered when interpreting the findings of this mini-review, consequently, we do not achieve a full-overview of the literature to develop a new tool. However, this mini review is useful to identify gaps in the knowledge for future research, e.g., the need for a youth specific food literacy tool. A strength is that the literature search was assisted by a librarian. This mini-review was carried out to provide an overview of tools used to measure food literacy and nutrition literacy as a sub-competence of food literacy in young people aged 16–24 years in the previous 5 years. This mini-review underlines that there is a need for continued effort to make comprehensive tools that measure the complete concept of food literacy in youth. However, this is challenging, as there is currently no clear consensus on how to measure food literacy in a youth population.

## Author contributions

SG-J and AM contributed to the conception and design of the study. SG-J performed the literature search and screening and wrote the first draft of the manuscript. AM wrote sections of the manuscript and supervised. All authors contributed to the manuscript revision, and read, and approved the submitted version.

## Conflict of interest

The authors declare that the research was conducted in the absence of any commercial or financial relationships that could be construed as a potential conflict of interest.

## Publisher’s note

All claims expressed in this article are solely those of the authors and do not necessarily represent those of their affiliated organizations, or those of the publisher, the editors and the reviewers. Any product that may be evaluated in this article, or claim that may be made by its manufacturer, is not guaranteed or endorsed by the publisher.
